# Validity in the Next Era of Assessment: Consequences, Social Impact, and Equity

**DOI:** 10.5334/pme.1150

**Published:** 2024-09-11

**Authors:** Benjamin Kinnear, Christina St-Onge, Daniel J. Schumacher, Mélanie Marceau, Thirusha Naidu

**Affiliations:** 1 Department of Pediatrics, University of Cincinnati College of Medicine, Cincinnati, Ohio, USA; 2 Department of Medicine, Researcher at the Center for Health Sciences Pedagogy, Université de Sherbrooke, Sherbrooke, Québec, Canada; 3 Department of Pediatrics, University of Cincinnati College of Medicine/Cincinnati Children’s Hospital Medical Center, Cincinnati, Ohio, USA; 4 School of Nursing, Université de Sherbrooke, Sherbrooke, Québec, Canada; 5 Department of Innovation in Medical Education, Faculty of Medicine, University of Ottawa, Canada; 6 Department of Psychiatry, University of KwaZulu-Natal, South Africa

## Abstract

Validity has long held a venerated place in education, leading some authors to refer to it as the “sine qua non” or “cardinal virtue” of assessment. And yet, validity has not held a fixed meaning; rather it has shifted in its definition and scope over time. In this Eye Opener, the authors explore if and how current conceptualizations of validity fit a next era of assessment that prioritizes patient care and learner equity. They posit that health profession education’s conceptualization of validity will change in three related but distinct ways. First, consequences of assessment decisions will play a central role in validity arguments. Second, validity evidence regarding impacts of assessment on patients and society will be prioritized. Third, equity will be seen as part of validity rather than an unrelated concept. The authors argue that health professions education has the agency to change its ideology around validity, and to align with values that predominate the next era of assessment such as high-quality care and equity for learners and patients.

Validity has long held a venerated place in education, leading some authors to refer to it as the “sine qua non” or “cardinal virtue” of assessment [1,2]. And yet, validity has not held a fixed meaning; rather it has shifted in its definition and scope over time. How will validity change as health professions education (HPE) assessment evolves? In this Eye Opener, we explore if and how current conceptualizations of validity fit the values in a next era of assessment that focuses on ensuring high-quality care for patients. Specifically, we explore what might be required for validity to support a world in which assessment is more socially accountable and equity-focused.

## A brief overview of validity and some contemporary conceptualizations

Validity conceptualizations in HPE have evolved over time. In 2017, St-Onge et al. [3] made explicit three different, co-existing conceptualizations of validity in the HPE literature: validity as a test characteristic, validity as an argument-based evidentiary chain, and validity as a social imperative (a conceptualization still nascent in HPE).

The first conceptualization, validity as a test characteristic, is strongly tied to measurement models, namely Classical Test Theory, Generalizability Theory and Item-Response Theory [4,5]. These theories and models aim to quantify measurement error and infer individuals’ “true” scores [4,6,7]. Reliability and validity are significantly intertwined, with the pursuit of a true score (Classical Test Theory), or a generalizability coefficient (reliability of score given a specified universe of generalization) [4]. Additional pursuit of score precision can be seen in Item Response Theory, which focuses on individual item-level difficulty [5,6]. In this view of validity, quantitative evidence to support an assessment score’s reliability, generalizability, or precision is highly valued. Validity is a characteristic attributed to a test, indicating that “it measured what it intended to measure” [8,9]. This conceptualization of validity still exists in HPE, most often with regard to sellable assessment products.

The second conceptualization, validity as an argument-based evidentiary chain, focuses on documenting the appropriateness of the interpretations and decisions made based on assessment data [3,10]. Two argument-based approaches have been predominantly imported into HPE, Messick’s unified theory of validity [11] and Kane’s approach to validation [12,13]. Authors that imported these approaches into HPE translated abstract validity conceptualizations into more concrete validation practices (e.g., Cook and Hatala [14], Kinnear et al. [15]). With the multi-faceted and complex programs of assessment that are increasingly found in competency-based education (CBE) [16], argument-based approaches allow for multiple, different types of evidence to be developed and integrated into fit-for-purpose arguments about the validity of assessment decisions. Validity as a social imperative (the third conceptualization) has grown out of argument-based approaches and, as we detail below, aligns well with the next era of assessment.

## The next era of validity in HPE assessment

The next era of validity will be shaped by broader forces and trends in HPE assessment. As a result, we believe validity will change in three related but distinct ways. First, HPE has already integrated the consequences of assessment decisions into validity conceptualizations, though consequences remain mostly unaddressed in real-world validation work. In the next era, consequences of assessment decisions will play a central role in validity. Second, the proliferation of CBE has foregrounded assessment’s role in social accountability. In the future, validity arguments will be more directly connected to impacts on patients and society. Third, equity considerations have become central in many HPE spaces. Similarly, equity will become central to future validity arguments. While consequences of assessment, social accountability, and equity are not novel concepts in HPE assessment, they are not central to most work on validity. Below we expand on how each will play a central role in the next era of validity.

### Focus on consequences of assessment decisions

Messick’s unified theory of validity brought with it the concept of different sources of evidence that can be sought to support the validity of assessment decisions [11]. One such source of evidence was the social consequence of test uses, which Messick called “consequential validity” evidence [17], now sometimes called “consequential evidence” [18]. Cook and Lineberry explored consequential evidence in HPE, describing it as “the impact, beneficial or harmful and intended or unintended, of assessment.” [18]. Consequences include impacts on learners, educators, programs, patients, and other systems and people. The concept of consequential validity evidence has gained acceptance in HPE, regularly appearing in HPE manuscripts describing the concept of validity and the process of validation [1,19,20,21,22,23,24]. Even the oft-cited Standards for Educational and Psychological Testing includes consequential evidence as important for validity arguments [10].

Cook et al. argue that “evidence of consequences is ultimately the most important source of validity evidence” [18]. The authors take a teleological stance, drawing an analogy with clinical diagnostic tests. Regardless of a diagnostic test’s sensitivity or specificity, its ultimate value will depend on consequences to patients, hospitals, and society. Similarly, while all sources of validity evidence have value, consequential evidence should be central to any validity argument. Despite this, consequential evidence is one of the least reported types of validity evidence. Across three systematic reviews on HPE validation work [25,26,27], consequential evidence was reported in only 5–20% of studies [18]. While one cannot say for certain why consequential validity evidence is relatively rare in HPE, contributing reasons likely include challenging study designs, limited resources for validation work, and vestigial preferences for more psychometric data such as reliability (i.e. internal structure) or criterion (i.e. relationship to other variables) evidence.

By making consequential evidence part of (or central to) validity arguments, we expand validity’s reach. Validation becomes more than ‘demonstrating that you are measuring what you think you are measuring’ to also include downstream effects of assessment. The next era of validity should include more widespread integration of consequential evidence into validity arguments. In the following sections, we explore how studying social impact and equity in assessment can provide meaningful consequential evidence.

### Evidence of the social impact of assessments

As noted above, consequential validity evidence includes impacts of assessment decisions on patients and society, and hence represents a form of social accountability. CBE, the predominant training philosophy in HPE in many countries, is rooted in social accountability [28,29]. Marceau et al. recently made explicit the concept of validity as a “social imperative” [30,31], in which validation is a mechanism to ensure that assessment decisions are linked to societal impacts. This view brings a deontological lens to consequential validity evidence by emphasizing HPE’s social contract with the patients it ultimately serves. To that end, the next era of validity will require evidence that assessment ensures trainees and graduates of HPE programs are providing high-quality care.

While the connection between HPE and patient outcomes is complex and non-linear [32], promising approaches are emerging to develop such consequential validity evidence. Clinical care measures that are seen as sufficiently attributable to individual trainees are being developed in multiple medical specialties [33,34,35,36]. Improving technology, such as haptics and artificial intelligence, could provide real-time assessment of procedural, communication, and teamwork skills [37]. Better understanding of interdependence of competence could unlock new ways of assessing team-based care outcomes [38,39]. All of these approaches can be integrated into current programs of assessment to bolster social accountability by connecting education to patient care.

### Centering equity in assessment validity

By recognizing consequential evidence as essential to validity arguments, we also make equity central to validity. We define equity in assessment as the opportunity to demonstrate and develop one’s knowledge, skills and abilities without negative influence by “structural or interpersonal bias related to personal or social characteristics of learners or assessors.” [40]. Equity goes beyond impartiality and includes efforts to ensure that each learner is afforded the resources and opportunities that they need to succeed, acknowledging that individuals need different types and levels of support and face different societal and system biases [41,42,43,44].

Equity is certainly not new to assessment, with scholars and advocates noting the many biases and injustices that have plagued HPE assessment for years [45,46,47,48,49]. Taking a sociocultural view, assessment has played a key role in creating and maintaining hegemony via control of patronage and access to educational and professional opportunities [50]. Performance on any particular assessment favors the dominant social order which influenced an instrument’s creation, while establishing what is accepted as truth and knowledge [50]. Viewed this way, assessment reinforces power structures while normalizing judgment. Thus, attending to equity is critical to promote fairness and justice for everyone impacted by assessment, particularly those who have been marginalized in a society.

Contemporary advocacy efforts such as #MeToo; Black Lives Matter; and advancing LGBTQ+, Feminist and Indigenous rights have brought equity to the fore of many discourses in HPE. Inequitable assessment is increasingly recognized as a driver of significant and tangible negative effects on learners that amplify and compound over time [45]. Equitable assessment should include choices of assessments that are inclusive of learners who require accommodations [41,51]. Current assessment accommodations often require learners to come forward, self-identify, and justify their requests [52]. However, education systems are rarely designed to help learners feel comfortable enough to do this [41,44]. Inequitable assessment also stands to harm patients by reducing the diversity of healthcare professionals that are available to serve diverse patient populations [53,54,55]. The next era of assessment brings a growing urgency to foreground equity in assessment [41,44,56,57], and validation practices should align with such goals.

Evidence of equity in assessment can be sought by examining the design of assessment tools (i.e. intrinsic equity), the learning environment (i.e. contextual equity), or the uses of assessment data to create equitable opportunities (i.e. instrumental equity) [46]. Onumah et al provide an example of how assessment systems can be designed with all three facets of equity in mind [58]. Equity also means programs should seek to understand how colonialism, racism, and Global North Euro-American principles have shaped HPE’s ideology and propagated inequities [47]. Including equity in validity arguments means that if assessment decisions are shown to worsen inequity for learners or patients, then we deem those decisions not valid.

Notably, we are not implying that equity is secondary or subordinate to validity, nor that equity is only important if examined through the lens of validity. We also do not believe that all of the richness, complexity, and nuance of equity initiatives can be captured within a validity argument. However, equity has long been treated as an afterthought in HPE assessment. By making equity a central part of validity arguments, it too becomes a ‘cardinal virtue’ of assessment. Therefore, in the next era of assessment, equity can function much like the concept of reliability – standing as a distinct concept while also being an integral part of validity arguments.

## Broad vs narrow conceptualizations of validity: choosing our ideology

HPE is not a monolithic group, and we do not expect that everyone will agree with our call to foreground consequences, social impact, and equity in assessment validity. Some validity scholars disagree with the assertion that consequential evidence should be part of validity at all, instead advocating that validity should focus only on construct representation, not the downstream impacts of assessment [59,60,61,62,63,64]. We are not implying that those scholars do not care about the consequences of assessment such as societal impact or equity. Rather, they see consequences as being different from validity, to be considered separately under categories such as “utility” or “acceptability”. The varying conceptualizations of validity reflect the many disciplines that comprise HPE (e.g. Psychology, Sociology, Measurement, Education) [65], and this diversity of viewpoints represents a strength for our field. Which view will predominate in the next era of assessment?

The wonderful news is that HPE has agency in such a choice. As Varpio points out, “Fortunately, ideology is maintained by our decisions and actions; therefore, we can change our decisions and thereby modify the ideology to work for us, not against us.” [66]. As the next era unfolds, we can align our conceptualizations of validity with the values underpinning our assessment work. As noted at the outset of the article, validity holds a long and tenured position as being the “sine qua non” of assessment. We believe consequences, social impact and equity deserve the same status in the next era or assessment.

A significant challenge will involve anticipating and identifying all relevant consequences of given assessments. When considering the equity consequences of assessment, where do we begin and end? Equity initiatives often range from efforts to improve equitable access to medical education, to ensuring diverse individuals have proper resources to succeed in training programs, to monitoring for negative downstream consequences of assessment decisions. Real-world decisions will be needed to determine where to focus energy and resources in developing validity arguments with a seemingly infinite amount of evidence to be collected. To navigate these discussions, a socio-constructivist approach to validity could be embraced to co-construct the argument to support the defensibility of these assessment choices. Or perhaps critical theory approaches can help ensure that we are scrutinizing who gets to decide what is equitable, whose voice is being valued, and why.

The next era of validity will require adopting a more inclusive perspective. Most, if not all, current authors and leaders in validity are from Europe and North America [67,68]. Thus, we must be mindful before transposing these validity conceptualizations and practices in other contexts and regions. Anecdotal evidence suggests that Global North customs and practices may not always transpose well in the Global South context. This should be further investigated. We can learn from the current and ongoing challenges of applying Global North conceptualizations in the Global South to challenge our assumptions about validity and validation and inform future development. We should also be open to the idea of validity expanding even further to include not just equity, but ideas such as social responsiveness and awareness. We have agency to determine where the boundaries of validity lie, how they can and should change over time, and which approaches best serve our learners and patients.

## Conclusion

The values of consequences, social accountability and equity will significantly influence assessment and validity in their next era. These values will undoubtedly challenge the current approach to validation and may require some to reconsider what falls under the purview of validity. A broader conceptualization of validity and validation that incorporates equity concepts in the purpose, design, and use of assessment data could contribute to assessments that are not just technically and psychometrically sound, but also socially accountable and equitable for learners and patients. Like so many required changes in assessment, changing our conceptualization of validity requires a shift in ideology. The next era in assessment has the potential to catalyze novel ways to develop, share, and evaluate validity arguments with impacts on our patients and learners at the center of what we do.
